# Letter from the Editor in Chief

**DOI:** 10.19102/icrm.2019.100808

**Published:** 2019-08-15

**Authors:** Moussa Mansour


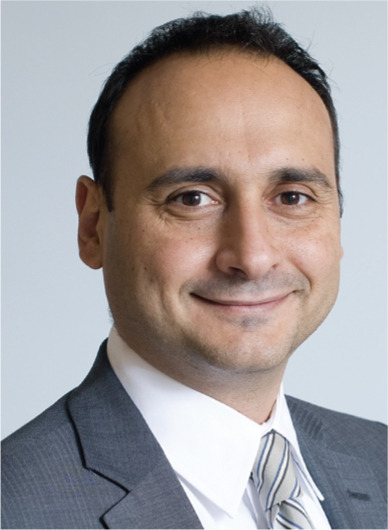


Dear Readers,

Atrial fibrillation (AF) is one of the most common known causes of stroke. Patients with AF are five times more likely to experience a stroke as compared with those without. Moreover, episodes of stroke caused by AF are generally more debilitating and deadly than those not associated with AF. As a result, stroke in the setting of AF has become a major clinical and economic burden.

Oral anticoagulants have been demonstrated previously to significantly reduce the risk of stroke in AF. The data supporting their use are robust and apply to all categories of anticoagulants including warfarin and novel anticoagulants. However, despite the abundance of data, more than 40% of patients who are candidates for oral anticoagulation are still not receiving these medications.

This issue of *The Journal of Innovations in Cardiac Rhythm Management* contains an article that highlights this concern, titled “Anticoagulation Patterns in Patients with Atrial Fibrillation Following Percutaneous Coronary Intervention in an Academic Center.”^1^ In this study, the authors analyzed the patterns of oral anticoagulation use in patients with AF who underwent percutaneous coronary intervention at a single center. They found that only half of the patients who met the indications for anticoagulation were actually given the drugs after their procedure. This finding, while very important, is not surprising given that anticoagulants are also underused in the overall population with AF.

The reasons for the underutilization of anticoagulants are not clear. Large population studies are needed to determine the reasons for not prescribing anticoagulation. One can expect that some patients have a legitimate reason for avoiding anticoagulation such as a prior intracerebral hemorrhage. However, another reason for not prescribing the medication may be a lack of knowledge, mostly on the part of the patient. Identification of the cause will help with the design of remedies to improve stroke prevention: patients at risk of bleeding may benefit from appendage closure, while, in others, education may help to convince the patient about the value of anticoagulation.

At a time when advanced pharmacological and nonpharmacological stroke prevention treatments are readily available, every effort should be directed to identify and target patients who are not currently being treated. Stroke resulting from diagnosed AF can be prevented to a large extent.

I hope that you enjoy reading this issue of the journal.

Sincerely,


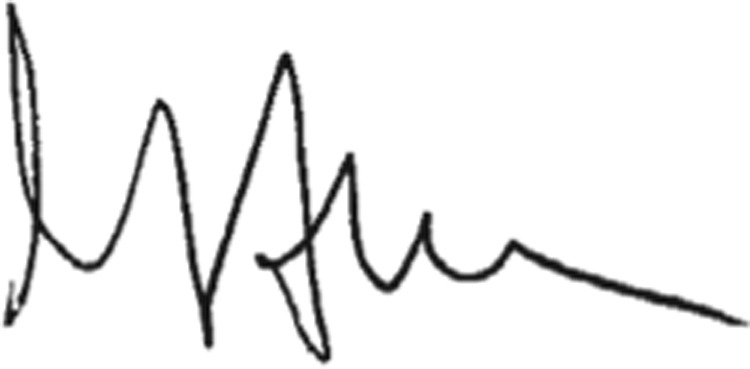


Moussa Mansour, md, fhrs, facc

Editor in Chief

The Journal of Innovations in Cardiac Rhythm Management

MMansour@InnovationsInCRM.com

Director, Atrial Fibrillation Program

Jeremy Ruskin and Dan Starks Endowed Chair in Cardiology

Massachusetts General Hospital

Boston, MA 02114
